# Edible Films and Coatings as Food-Quality Preservers: An Overview

**DOI:** 10.3390/foods10020249

**Published:** 2021-01-26

**Authors:** Elsa Díaz-Montes, Roberto Castro-Muñoz

**Affiliations:** 1 Unidad Profesional Interdisciplinaria de Biotecnología, Instituto Politécnico Nacional, Av. Acueducto s/n, Barrio La Laguna Ticoman, Ciudad de México 07340, Mexico; elsadimo123@gmail.com; 2 Tecnologico de Monterrey, Campus Toluca, Avenida Eduardo Monroy Cárdenas 2000, San Antonio Buenavista, Toluca de Lerdo 50110, Mexico

**Keywords:** polysaccharides, lipids, proteins, edible films, edible coatings, conservation, quality, foods

## Abstract

Food preservation technologies are currently facing important challenges at extending the shelf-life of perishable food products (e.g., meat, fish, milk, eggs, and many raw fruits and vegetables) that help to meet the daily nutrient requirement demand. In addition, food preservation has gone beyond only preservation; the current techniques are focused on the fulfillment of two additional objectives, the suitability of the used processes and generation of environmentally friendly products with non-presence of any side effect on health. Moreover, they are also looking for additional nutritional properties. One of these preservation protocols deals with the use of edible films and coatings. Therefore, this review shows an overview of synthetic materials (e.g., glass, aluminum, plastic, and paperboard), as well as the regulations that limit their application in food packaging. Further, this review releases the current-state-of-the-art of the use of films and edible coatings as an alternative to conventional packaging, providing the main features that these biodegradable packaging should meet towards specific uses for the conservation and improvement of various food products. Herein, particular attention has been paid to the main used components (e.g., biopolymers, additives, bioactive, and probiotic components), manufacturing methods (for edible films or coatings) and their application to specific products. In addition, an outlook of the application of edible films and coatings as quality indicators of perishable products is shown.

## 1. Introduction

**Figure 1:**
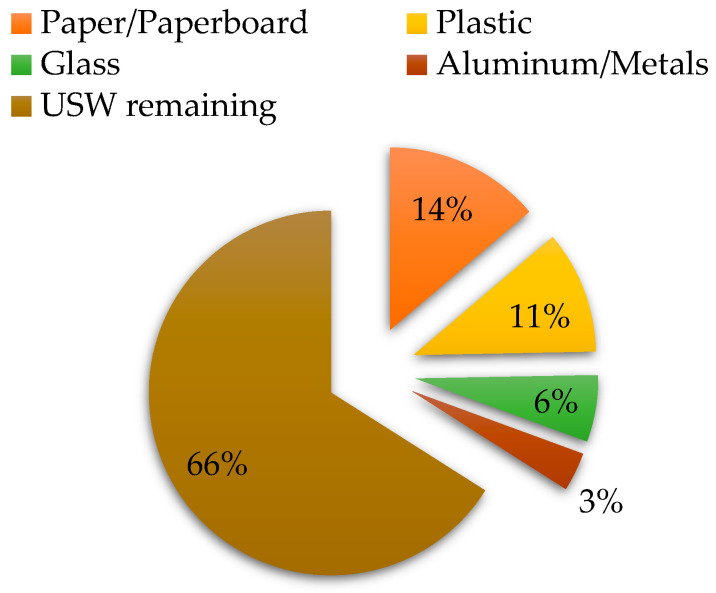
Distribution of packaging materials as part of the generation of urban solid waste (USW) [4].

The packaging is likely the most important method for food preservation due to protects, preserves and provides the needed information about the product, while allows the product commercialization and distribution [1,2]. The packaging’s characteristics depend on the food product that is desired to be protected. To date, different materials have been employed as packaging materials, such as paper, cardboard, metal, glass, plastic, among others [3]. However, it is likely that this traditional preservation method is the one that produces large quantities of urban solid wastes (USW). For instance, according to the most recent data (in 2018) provided by the Ministry of Environment and Natural Resources (SEMARNAT) of Mexico, production of about 102,895 USW ton per day is generated, which corresponds to paper, cardboard, glass, and some metals (aluminum). Figure 1 illustrates the percentages that correspond to each material [4]. Despite the campaigns promoted by the Secretary of the Environment (SEDEMA) regarding integral waste management, only 10% of the total USW is recycled [4,5]. Therefore, most of the packaging materials have unique use before being discarded.

On the other hand, companies, which prefer to recycle the materials, are also facing the issue of classification since most of the packages are constituted by a mixture of materials with different characteristics. At this point, the recovery, selection, cleaning and reprocessing of materials make the recycling a complicated and expensive task [2]. For this reason, renewable raw materials have been deeply explored in recent years at aiming to minimize pollution problems through alternative biodegradable packaging [6]. In this way, packaging based on biodegradable materials have emerged and evolved as an option to replace traditional materials that cannot be recycled. Such biodegradable materials can protect the product while their production, recycling, and degradation are relatively easy [1]. Generally, most of the biodegradable packaging implies the use of environmentally friendly polymeric materials aiming for the preservation of quality and extending the self-time of the minimally processed products, such as fruits and vegetables [7,8]. Herein, the goal of this review paper is to provide an overview of the current-state-of-the-art of the use of edible films and coatings in different foods, paying special attention to the main used components (e.g., biopolymers, additives, bioactive, and probiotic components), manufacturing methods (for edible films or coatings) and their application to specific products. Moreover, this review provides the main features that biodegradable packaging should meet to be considered as edible films and coatings towards specific uses for the conservation and improvement of various food products.

## 2. Food Packaging and its Role in Food Preservation

Once foods are minimally or fully processed, the packaging is becoming the most important step due to allows their transportation from the factories to the point of sale or distribution. Thereby, such material contributes to maintain most of the physicochemical, functional, and organoleptic characteristics of the food [2,9]. In addition to this, the packaging must not interact with the product and protect it from external damage of chemical, physical, and biological type [2]. Chemical damage includes exposure to gases, moisture and light; physical damage refers to any damage caused by any shock or vibration; and biological damage is caused by the action of pathogens, insects, animals, or the senescence of the food itself [9]. The next section addresses the overview of the different food packaging materials, including plastic, metal, glass, paper, among others, towards the preservation of the foods.

### 2.1. Conventional Food Packaging Materials

Plastic is the most known packaging material, and many petrochemical-based materials are currently used due to their availability at a relatively low cost. However, environmental conservation regulations have strongly restricted their use as packaging materials since they are not fully recyclable or biodegradable [10]. In the case of paper, it is used as packaging due to the fact that it comes from a biodegradable matter; nevertheless, being in contact with food loses its physical appearance and prevents its protection. This is one of the main reasons to combine it with other materials, such as plastic and aluminum. Unfortunately, paper loses its biodegradable effect and the feature to be recycled [11].

Glass is likely the oldest material used as packaging. It is chemically inert and odorless making its use very wide. Nowadays, the packages based on the glass are thin and resistant to sterilization treatments at high temperatures and pressures [9]. Metals are the most versatile material in all forms of packaging since it is highly resistant and fully recyclable. Aluminum and steel are found as the most common metals. Aluminum has the advantage of being moldable to the product, while steel is only used as a container. In general, metal gaskets protect against moisture, air, odors, and microorganisms [9].

Table 1 summarizes the type of packaging based on such materials and their advantages and disadvantages as barrier types. Their selection depends specifically on the type of food or product to be protected. For example, jellies and sauces without any preservatives are mostly packed in glass containers because the protection against biological agents is almost ensured, allowing their preservation for a long time [9]. The canned products are mostly packaged in aluminum containers to avoid odor exchange and microbial contamination [2,9]. While products of fast consumption due to their short life, such as milk, bread and cookies, are usually packed in plasticized cardboard, being sufficient for protection in short times [2].

### 2.2. Food Packaging Laws and Regulations

There are laws that regulate the quality control of packaging in terms of their interaction with the food products (packaging–product relationship). These regulations are complex due to the diversity of specific packaging, variety of materials (such as paper, glass, and plastic), presentations (e.g., boats, boxes, and bags), aggregates of presentations (e.g., inks, adhesives, and seals) and the characteristics of the food products (e.g., moisture, fat or alcohol content, pH, and freshness) [12]. The Food and Drug Administration (FDA) has mentioned that any possible contamination packaging-product may be associated with the recycling of the packaging material, exposed to any harmful substance from packaging solutions, or generated during treatments (e.g., thermal or chemical). Likewise, such substances can be contained for a long time in reused packaging material [13]. Although, there are standards that specify the maximum acceptable levels of chemical contaminants being in contact with the foods [13]. Herein, the packaging should meet five basic requirements to be commercially available: (i) the packaging should not display any human health risk, (ii) the packaging should not change the physicochemical composition of the food, (iii) the packaging should not change the organoleptic features of the food, (iv) the packaging must be manufactured and treated according to good manufacturing practices, and (v) the packaging must not present misleading information about the contained product [12].

On the other hand, the legislation established by the International Organization for Standardization (ISO) also deals with regulations that involve the production, distribution, and use of packaging materials, such as ISO 18604:2013(E). Such regulations establish the requirements that the different food packaging materials should meet in order to be collected, processed, and recycled as a new feedstock [14]. Although these norms help to control the quality of food products through adapting the packing materials; there are also regulations that rule the environmental aspect of the waste production from packaging [15], which have restricted the use of packages that contribute directly or indirectly to the pollution of the flora and fauna. However, the total elimination of packaging is perhaps impossible; this is due to the food needs always feasible protection during its distribution that allows them to be maintained until consumption [9]. Whereas the European Union legislation covers all materials that may be in direct or indirect contact with food, for example, production machinery, kitchen utensils involved in filling and containers and packaging used for distribution (regulation EC 10/2011). This regulation includes specific specifications on the use of active and intelligent packaging (regulation EC 1935/2004) since they can only release substances accredited as food additives and must be accompanied by a declaration of conformity [16]. For this reason, by considering specific products, edible films and coatings have become a latent and promising alternative to preserve and even enhance the quality of the foods during their processing and storage [17]. Thereby, the following sections of this review provide a critical overview in applying edible films and coatings for food preservation, addressing the following aspects: main components and their properties, feasible protocols and techniques for coating fabrication, and applications and the most recent advances in the field.

## 3. Edible Films and Coatings as Packing Materials

### 3.1. Characteristics of Edible Films and Coatings

An edible film or coating is any material with a thickness of less than 0.3 mm [18], which is formed from a combination of biopolymers and different additives (Section 3.2) dispersed in aqueous media [19,20,21]. Some authors use the terms of edible film and coating interchangeably; however, others consider that there is a distinction due to the techniques of incorporation into the food product [22]. The edible coating is formed directly on the food, while the edible film is previously made and then adhered to the product [22,23]. Despite this, in both cases, rigid matrices with similar characteristics are formed [6,24].

**Figure 2:**
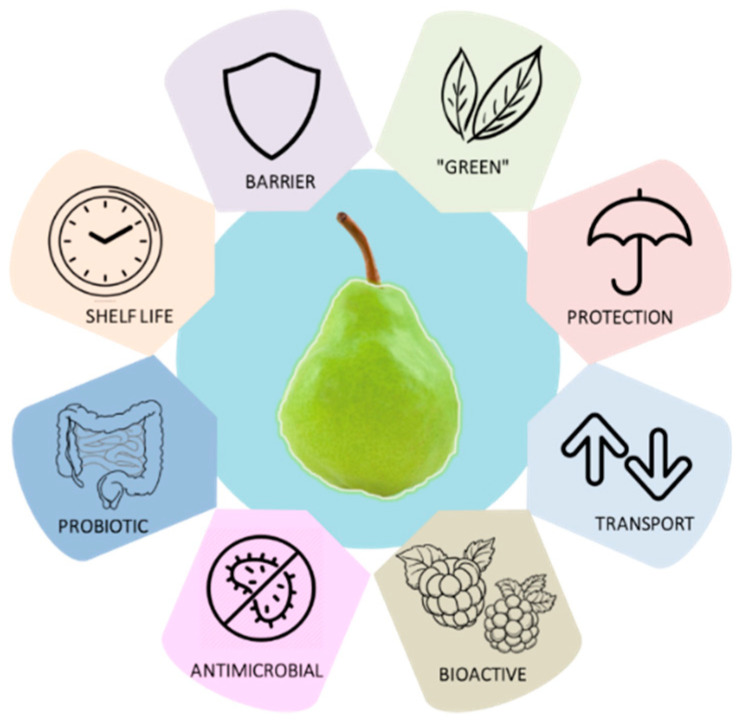
Scheme illustrating the main characteristics of edible films and coatings.

Figure 2 illustrates the main characteristics that edible films and coatings can present: (i) protection against UV light [17]; (ii) transport of solutes (e.g., salts, additives, and pigments), water vapor, organic vapors (e.g., aromas and solvents), and gases (e.g., oxygen, carbon dioxide, nitrogen, and ethylene) between food and the atmosphere [17,25]; (iii) barrier against mechanical damage (e.g., dents or cuts) [22]; (iv) increase the shelf-life of the product [25]; (v) bioactive components (e.g., antioxidants) [26,27]; (vi) antimicrobial effect against bacterial reproduction and fungal contamination (e.g., silver nanoparticles) [26,28]; (vii) healthy microorganisms (e.g., probiotics) that confer benefits to the consumer; and (viii) biodegradable natural materials [22].

### 3.2. Materials of Edible Films and Coatings

In addition to similar characteristics (Section 3.1), edible films and coatings are often evaluated for their mechanical properties, such as elasticity modulus (EM), elongation at break (E), and tensile strength (TS) [29,30,31], which refer to their elasticity and rigidity, and the force necessary to break them [32]. Further, they display similar mass transfer phenomena (i.e., permeation, adsorption, and diffusion), which is related to the transport of solutes between food and the atmosphere [29]. However, both mechanical properties and mass transfer phenomena are influenced by the type of material and manufacture protocol that allows the generation of different structures of biopolymeric matrices [33,34,35,36]. Table 2 enlists the most used biopolymers and additives in the production of edible films and coatings, together with their properties and functionality in packing.

Starch is considered the universal biopolymer for bio-packaging, which has been widely used for decades [48], due to its characteristics and gelatinization properties [29]. Alginate is another important biopolymer that displays the ability to form hydrogels and encapsulation barriers [49,50]. However, chitosan has recently attracted attention for the elaboration of edible films and coatings [51] due to their properties as a gelling agent and their chemical (it could form hydrogen bonds and hydrophobic interactions) and biological (its biocompatibility, biodegradability, and bioactivity) properties [52,53]. While other authors have selected the use of other natural components for the formulation of packaging, including proteins (e.g., collagen and protein isolates) [54,55], lipids (e.g., canola oil and cinnamon bark oil) [56,57], among other unconventional materials (e.g., smooth-hound protein and papaya puree) [58,59] to produce bio-packaging with targeted characteristics.

On the other hand, the role of additives (e.g., plasticizers or stabilizers) in the formulation of edible films and coatings is to modify the mechanical properties (to ideally increase E and decrease TS and EM) and mass transfer phenomena [6,29]. Furthermore, the incorporation of antioxidant, fungicidal, or microbial additives allows obtaining bioactive bio-packaging [45,60]; which will be discussed in detail (Section 4).

### 3.3. Disperse Systems Forming Edible Films and Coatings

**Figure 3:**
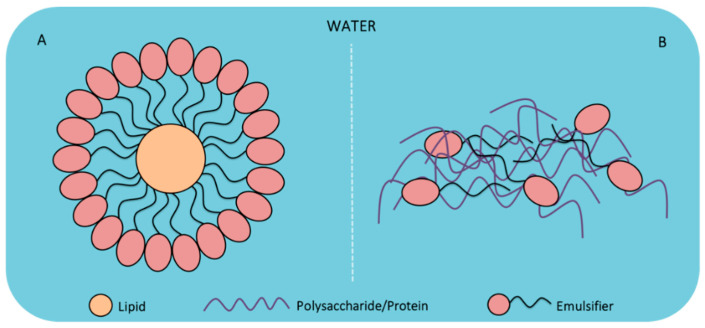
Main types of dispersed systems generated based on biopolymers, (**A**) emulsion oil/water (o/w) and (**B**) colloidal dispersions.

The biopolymeric materials used for the formulation of bio-packaging are incorporated in different ways due to their glucidic (i.e., polysaccharides), proteinic or lipidic nature; creating dispersed emulsion-type (i.e., based on lipids) or colloidal systems (i.e., based on polysaccharides or proteins) [61]. Figure 3 outlines the two types of systems that can be formed for the generation of edible films and coatings.

Emulsions are systems composed of liquid or semi-liquid substances that are immiscible to each other, e.g., an oil and aqueous phase that can be merged by means of an emulsifying agent. The emulsifying agent generally possesses a hydrophilic and a hydrophobic zone, displaying an affinity to polar and non-polar sites [26,62]. Thus, emulsions can be classified in two types depending on the proportions of their phases, e.g., oil/water (o/w) or water/oil (w/o) because the dispersed phase corresponds to the second component, being in lower concentration in the emulsion [61,63]. In the formulation of edible films and coatings, the o/w systems are preferred (Figure 3A) since they are thermodynamically more stable and they can dissolve lipophilic antimicrobial components (e.g., plant essential oils) and bioactive components (e.g., fatty acids, carotenoids, antioxidants, phytosterols, or quinones) [26].

Colloid systems are polymeric systems that are made up of polysaccharides or proteins dissolved in an aqueous phase [61]; they form a dense matrix that can protect active components (e.g., antioxidant and antimicrobial agents) [64] and allow their controlled release in the matrix [65]. Due to the hydrophilic nature of polysaccharides and proteins, colloidal systems are mostly used for the development of edible films and coatings since they can transport and protect a large number of molecules that act as additives (e.g., essential oils) [66,67] and probiotics (e.g., lactic acid bacteria) [68,69]. Colloidal systems do not form a matrix with an ordered grouping in their polymeric components (Figure 3B) due to the fact that different types of interactions (e.g., ionic, hydrogen bridges, or electrostatic interaction) can be produced according to the type of biomaterial (i.e., protein or polysaccharide); therefore, they tend to which generate matrices with varied characteristics [70,71].

**Figure 4:**
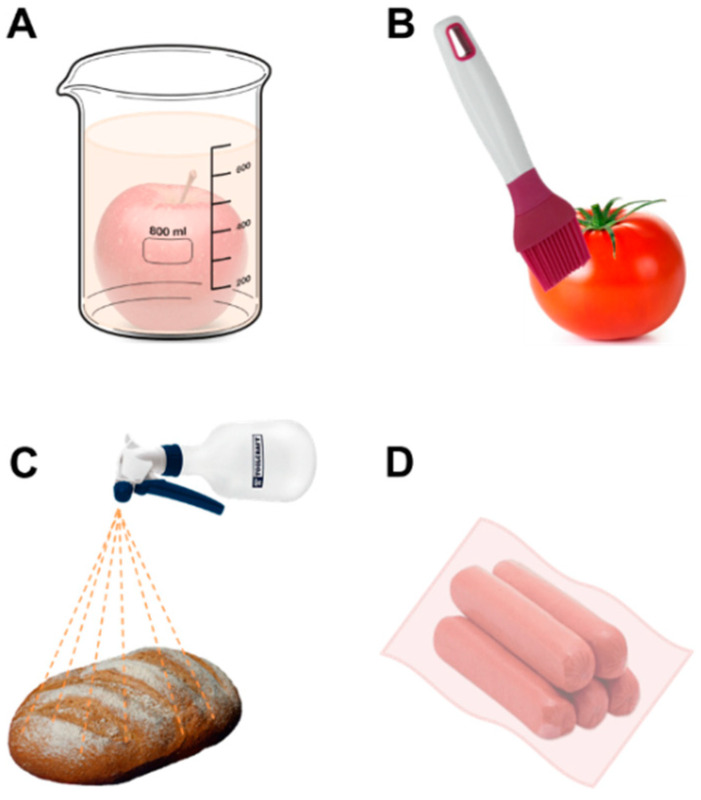
Main techniques used for food coating. (**A**) Dipped; (**B**) Spread; (**C**) Sprayed; (**D**) Wrapped.

The dispersed systems must be incorporated/coated into the product to dry (or vice versa) and subsequently generate a rigid matrix that will act as an edible film or coating. This will strictly depend on the type of application protocol [72]. The most used application techniques are (A) dipped, (B) spread, (C) sprayed, and (D) wrapped, as illustrated in Figure 4. The edible coating formulations are added and dried directly on the surface of the food (Figure 4A–C), while the edible film formulations are poured into a mold and dried to later be incorporated into the product (Figure 4D).

## 4. Recent Advances in Edible Films and Coatings

### 4.1. Edible Films as Functional Bio-Packages

According to the definition given by Kris-Etherton et al. [73], a bioactive component is any constituent contained in small amounts in food which can display effects on the health after their consumption. Epidemiological studies have analyzed different bioactive molecules (e.g., flavonoids and phytoestrogens), which in fact have been recognized by their antioxidant, antimutagenic, anti-inflammatory, anti-cancer, apoptotic, and anti-cholesterol effects [74,75,76,77,78,79]. Thus, various researches have been specifically focused on incorporating a wide variety of these bioactive compounds into edible films, as enlisted in Table 3.

For example, several authors have evaluated the antioxidant capacity of edible films with phenolic compounds extracted from various sources [85,86,89]. Nogueira et al. [85] determined that the antioxidant activity of blackberry powder is related to its anthocyanin content, and it was maintained when added to arrowroot starch edible films; furthermore, the water solubility, water vapor permeability, TS and E properties were improved with the addition of the powder, while the color and flavor characteristics of the blackberries remained. Rodsamram and Sothomvit [86] elaborated edible films based on coconut protein and coconut water, which presented antioxidant activity given by phenolic compounds of coconut; also, the brown coloration of the edible films exerted a barrier towards UV light. While Assis et al. [89] extracted and encapsulated β-carrot carotenes in a cassava starch matrix, resulting in edible films with antioxidant activity and improved solute transport. In these studies, the antioxidant effect was given by the action of bioactive substances that cause a significant delay in the oxidation of the substrate, and also the inhibitions of reactions involving free radicals [104].

Other authors have extracted oils from various sources, which have displayed antimicrobial activity; for example, Abdel Aziz et al. [67] incorporated castor oil to an alginate matrix to generate edible films with an inhibitory effect against *Staphylococcus aureus*, *Bacillus subtilis*, *Salmonella typhi,* and *Escherichia coli*. The greatest effect was noticed when increasing the concentration of castor oil due to an increase in the hydrophilic character of the edible films by the hydroxyl groups of edible oil, which easily dissolve the membrane cell and provoke the uncontrolled transport of substances into the bacteria [105,106]. Alvarez et al. [100] generated edible films of citrus peel pectin with oregano essential oil that exerted an effect against *Chromobacterium violaceum* by inhibiting cell communication due to the action of oregano essential oil [107]. Similarly, Alboofetileh et al. [99] used different essential oils (i.e., clove, cumin, caraway, marjoram, cinnamon, and coriander essential oils) in edible films based on alginate and montmorillonite. Thanks to the presence of the oils, the films showed antimicrobial activity against *Escherichia coli*, *Staphylococcus aureus,* and *Listeria monocytogenes* [99]; however, marjoram essential oil presented the highest inhibition due to the control of cell growth; in addition, the biopolymeric matrix formed between alginate and montmorillonite controlled the release of the oils, maintaining continuous inhibitory effect [108].

Some other biologically active compounds, such as organic acids (e.g., acetic acid, benzoic acid, sodium benzoate, and sorbic acid), peptides (e.g., nisin), and enzymes (e.g., lysozyme), have been incorporated into edible films and coatings for their antimicrobial action [109]. The antifungal effect and low toxicity (for the consumer) of natural components, such as citrus plants (e.g., lemon) or essential oils (e.g., cinnamon, clove and oregano essential oils), have also been demonstrated [110]. For example, the increasing of cinnamaldehyde concentration in chitosan-graph-based edible films has proven the antifungal properties against *Penicillium italicum* and *Rhizopus stolonifera* [103]. In addition, additives (i.e., cinnamaldehyde) improved the mechanical properties in terms of EM, E and TS testing. Tarazona et al. [102] also evaluated cinnamaldehyde and other additives (i.e., linalool, isoeugenol and citral) in edible films of ethylene-vinyl alcohol copolymer. The results showed different antifungal activities against *Aspergillus steynii* and *Aspergillus tubingensis*, but the effect was greater with the presence of cinnamaldehyde since there was a total inhibition of fungi [102].

Bioactive components are able to concurrently display several properties, which may produce a synergistic effect; for example, Ounkaew et al. [92] and Wei et al. [91] analyzed the antioxidant and antimicrobial capacity of two different edible films with incorporated organic acids. Ounkaew et al. [92] manufactured edible films based on cassava starch, extracted spent coffee ground and citric acid; which exhibited antioxidant capacity and inhibitory effect against *Escherichia coli* and *Staphylococcus aureus* given by the synergistic effect between the biopolymer and additives, together with the increasing content of citric acid. While Wei et al. [91] embedded lysozyme enzyme and ascorbic acid in zein-based edible film. The authors reported that higher enzyme concentrations resulted in better antimicrobial properties against *Listeria innocua* and *Micrococcus lysodeikticus*), while the increase of organic acids improved the antioxidant capacity of the edible films [91]; in addition, a synergistic effect in flexibility and mechanical properties was seen between zein, lysozyme and ascorbic acid.

### 4.2. Coatings as Pathogen Inhibitors in Food Models

Most of the characteristics of edible films and coatings are relevant; however, the biological protection of food is one of the most important since it directly affects the shelf-life of the product [111]. Therefore, it is necessary to inhibit or eliminate bacterial or fungal microorganisms (as well as their derivatives) that can cause or accelerate putrefaction in food due to the action of their enzymes and by-products produced from their metabolism (e.g., gases) [112].

**Figure 5:**
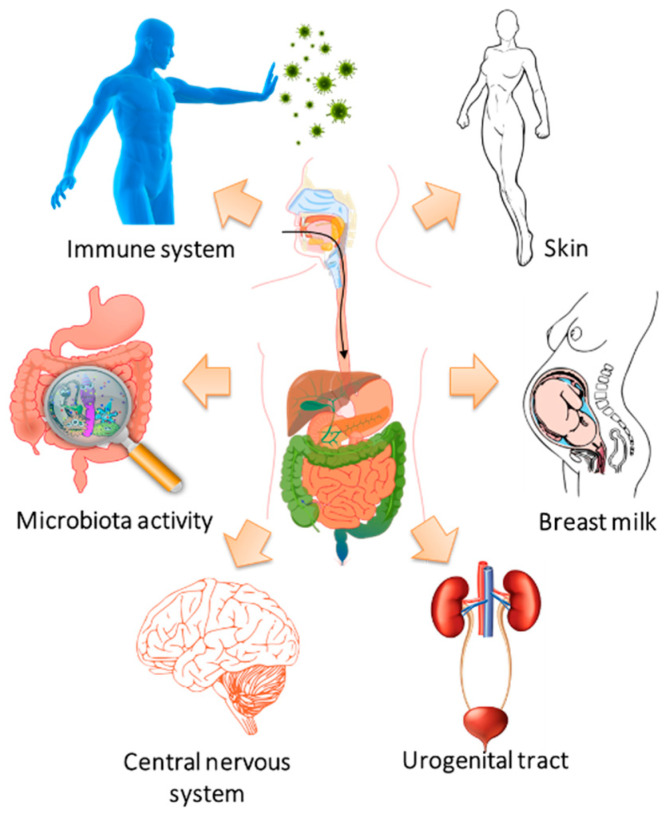
Schematic representation of the health benefits after probiotic consumption [113].

The lactic acid bacteria are generally recognized as safe (GRAS) and there are many kinds of research that show the beneficial effects when acting in the consumer’s gastrointestinal tract (Figure 5) [113]. Thus, some authors have also focused on evaluating the inhibitory effect of edible films with Lactic acid bacteria (LAB) and fungi activities against pathogenic microorganisms applied in food models, as summarized in Table 4.

For example, Aloui et al. [117] inoculated the *Wickerhamomyces anomalus* in two different edible films based on alginate and locust bean gum, and subsequently covered oranges finding that the bacteria had greater stability in the alginate matrix; in addition, alginate edible film managed to inhibit *Penicillium digitatum* and kept the fruit viable over 13 days. Parafati et al. [116] also inoculated *Wickerhamomyces anomalus*, *Metschnikowia pulcherrima* and *Aureobasidium pullulans* in mandarins coated with edible carob gum edible films; the findings show a greater inhibitory effect with *Metschnikowia pulcherrima* against *Penicillium digitatum* and *Penicillium italicum*.

Other studies of microorganisms incubate in coatings for the control of pathogenic fungi, as reported by Marín et al. and Fan et al. [115,121]. The first study evaluated several edible films (i.e., hydroxypropylmethyl cellulose, sodium caseinate, pea protein and corn starch) to serve as a support for *Candida sake* and later coat grapes to protect against *Botrytis cinerea* [115]; although all biopolymers were adequate to inhibit the pathogen and maintain the survival of *Candida sake*, the authors recommended the use of sodium caseinate and corn starch since they represent the lowest cost. In the second study, Fan et al. [121] used alginate edible film containing *Cryptococcus laurentii* to coat strawberries, the authors reported that the microorganism remained viable, and the edible films significantly reduced mold and improved the quality and physical appearance of the fruits.

### 4.3. Coatings as Probiotic Carriers in Food Models

Probiotics are a type of bioactive compounds with specific health benefits [122]. According to the definition in 2002 given by the Food and Agriculture Organization of the United Nations/World Health Organization (FAO/WHO), probiotics are “live microorganisms which, when administered in adequate amounts, confer a health benefit on the host” [123]. LAB of the genus *Lactobacillus* have been widely studied for their probiotic properties since they play an important role in preventing the deterioration of the microbiota and in the inhibition of pathogenic microorganisms (Table 4) at the oral cavity and colon [124]. In addition, there is evidence that relates the metabolic activity of LABs with the control of bacterial pathogens and fungal agents [113]; specifically, the authors associate the inactivation of pathogens by the effect of organic acids (e.g., lactic and acetic acids), carbon dioxide, ethanol, peptide compounds, and enzymes, that are produced within LABs metabolism [125]. Other authors relate the decrease in pH with the inhibitory effect because it generates an environment competition between the substrates of LABs and pathogenic microorganisms [113,126]. For this reason, the most exhaustive studies in probiotic evaluation have been carried out in the incorporation of microorganisms in edible films and coatings, rather than on their inhibitory efficacy against external pathogens (due to contamination of the environment). Figure 5 presents an overview of the main systems and organs benefited by the consumption of probiotic microorganisms, highlighting (i) the immune system: inflammatory control is maintained; (ii) the microbiota: the proliferation of pathogenic microorganisms, such as *Clostridium difficile* and *Helicobacter pylori*, is regulated; (iii) the nervous system: brain functions are modulated; (iv) the urogenital tract: urogenital infections are fought; (v) the placenta: probiotic microorganisms are transmitted to the fetus; and (vi) the skin: allergies and atopic dermatitis is helped [113].

The main purpose of stabilizing or keeping probiotics viable is that once ingested, they can withstand the conditions of gastric juices and intestinal fluids; in this way, they can be dosed periodically to carry out their probiotic effect [72,113]. Therefore, Gbassi et al. [127] studied the viability of *Lactobacillus plantarum* encapsulated in alginate and subsequently introduced in an edible film of whey protein under gastrointestinal conditions (pH 1.8 at 37 °C); as a result, the probiotics remained viable until 180 min. This study is relevant due to the simplicity of the matrix and its efficiency together with the costs of alginate and whey protein [127].

Importantly, the main objective of a probiotic embedded in edible films is to study and evaluate their viability within the matrix, as well as its interaction with the coated food and its probiotic activity. Table 5 presents different studies in which edible coatings were applied to food products, monitoring the viability of the probiotics directly on the product.

The viability of microorganisms varies when the edible films are being individually characterized in a specific product and stored under different conditions (e.g., temperature and relative humidity). For example, Soukoulis et al. [131] evaluated the survival of *Lactobacillus rhamnosus* in an alginate/whey protein matrix that covered bread. They achieved to maintain the LAB stability for seven days at 25 °C. Compared with their previous study [131], the authors found out that the viability time of *Lactobacillus* decreased considerably (up to 93%) when the bread was stored at 4 °C; but the bacteria viability increased up to 99 days when the matrix was not applied on the bread [134].

The difference in viability time of *Lactobacillus plantarum* can also be compared when stored and treated under different conditions; as reported by Tavera-Quiroz et al. [130] who incorporated *Lactobacillus plantarum* in a methylcellulose matrix to coat apple baked snacks and maintain their viability up to 90 days in simulated in vitro gastric conditions (two stages: pH 2.5 and 7.5, and 37 °C). Gbassi et al. [127] used a whey protein matrix and similar gastric conditions to Tavera-Quiroz et al. [130], maintaining the *Lactobacillus plantarum* viability for 180 min.

In particular, López De Lacey et al. [133] pointed out the importance of conserving fresh products, such as fish, since they are highly perishable products and susceptible to the development microorganisms and contamination [12,118]. The authors were able to extend the shelf-life of Hake fish up to 15 days at refrigerated conditions (4 °C) [133], at this point, *Lactobacillus acidophilus* and *Bifidobacterium bifidum* were deposited in a coating of gelatin, sorbitol, and cysteine.

### 4.4. Edible Films and Coatings as Food Preservatives

**Figure 6:**
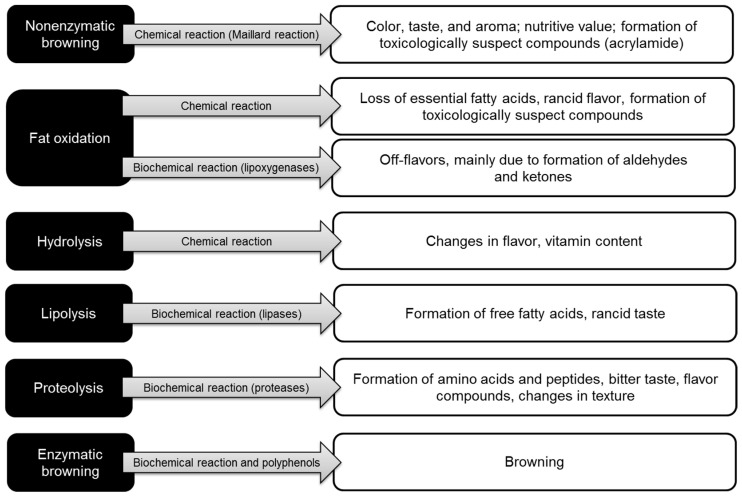
Chemical/biochemical reactions in foods affecting their quality [135].

**Figure 7:**
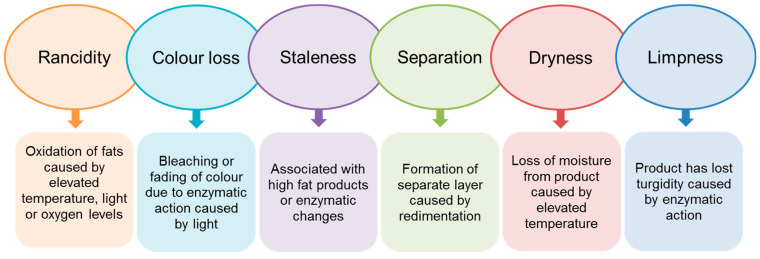
Non-microbial spoilage physical defects in foods [136].

Currently, the food industries have a duty to offer to the consumers fresh, pleasant, good quality food with beneficial properties for health [8]. However, the challenge of meeting consumer demands is very demand since there is no food that can remain in optimal conditions and maintain its properties permanently due to the natural deterioration generated by chemical, and biochemical reactions and physical changes [135]. Figure 6 and Figure 7 illustrate the different biochemical and physical defects developed in foods, respectively. Commonly, observed spoilage changes include unpleasant odors, rancidity, darkening, softening of the texture, and loss of nutrients and vitamins.

Food spoilage is influenced by oxygen availability, temperature, relative humidity, water content, and pH [137]. For this reason, besides the characteristics of the edible films and coatings themselves, it is of great interest to maintain the characteristics of the food, including: (i) preservation of microbiological parameters in accordance with established laws; (ii) preservation of nutritional content; and (iii) preservation of physical and sensory characteristics (e.g., smell, taste, and texture); which together extend the shelf-life of the product [136].

Table 6 reports different development works aiming at extending the shelf-life of plenty fruits, vegetables, animal, and dairy products; where the edible film/coating influenced positively the chemical, biochemical and physical parameters, minimizing the food spoilage, and thus increased the shelf-life of the food product.

According to the relevant findings reported by the research community, the maturity in fruits and vegetables and the mold and microbial growth can be delayed, preserving specific properties such as texture, freshness, vitamin C content and nutritional quality, as well as conferring new biological activities (e.g., antioxidant activity) depending on the types of bioactive solutes incorporated in the edible films and coatings. In animal and dairy products, the edible films and coatings allowed to maintain the bioactive components of the product itself and the sensory characteristics; the antimicrobial, antifungal and antioxidant activities, and shelf-life were also improved; while in bakery products, the moisture content decreased and the shelf-life increased.

## 5. Concluding Remarks

This review has compiled and analyzed the most recent studies about the application of edible films and coatings in a wide type of foods. Different types of materials have been used in manufacturing packing for the preservation and improvement of food products, emphasizing the bio-polymeric materials that have been used to form new barriers to directly protect the product. Furthermore, specific additives need to be incorporated to improve the physical characteristics and mechanical properties of the resulting packing. Today, as a current trend in the field, bioactive compounds and microorganisms (like probiotics) are added into sustainable packings to extend the functionality and nutrition of perishable and natural foods. The main application techniques that differentiate edible films from edible coatings were also shown, which influence together with the formation materials, the product quality, shelf-life, maturation, darkening effect, and the inhibition of pathogens.

To finalize, bio-packaging has demonstrated to meet the requirements for the protection of minimally processed foods and their use suggests an economic saving related to the loss of food due to natural maturation, managing to extend the shelf-life of the product. Depending on the biomaterials used and the types of biologically active compounds, specific properties, such as sensorial, physicochemical and nutritional characteristics, in coated products can be improved. However, there are still many biopolymers (e.g., zein) and additives with good characteristics to form edible films and coatings that have not been explored in detail, which may promise successful insights into the protection and preservation of food products.

## Figures and Tables

**Table 1:** Some advantages, disadvantages of the conventional packing materials [2,9].

Packing Material	Advantage	Disadvantage	Barrier Type	Food Type
Glass	Recyclable materials	Susceptible to breakage	Protection for chemical and biological agents, physical damage, and odors	Sodas pop Jellies Sauces
Metal	Recyclable materials	Expensive compared to other packing materials	Protection for chemical and biological agents, physical damage, and odors	Preserves Juices Fish
Plastic	Cheap materials	Association with other packaging materials	Permeability of gases and vapors	Sodas pop Water Bread
Paperboard	Biodegradable materials	Association with other packaging materials	Protection from physical damage, abrasions and crushing	Milk Cookies Eggs

**Table 2:** Main materials used and functionality in the manufacture of edible films and coatings [6,37,38,39,40,41,42,43,44,45,46,47].

Materials	Examples	Properties	Function in Edible Films and Coatings
***Biopolymers***			
Polysaccharides	Starch Cellulose Pectin Gums Chitosan Agar Alginate Dextran	Thickeners Gellants Emulsifiers Stabilizers Coating	They form the base structure of a solid polymer matrix.
Proteins	Gelatin Casein Whey protein	Gellants Thickeners Stabilizers Foaming	They help in the transport of antimicrobials and antioxidants. They control the transport of gases (mainly oxygen).
Lipids	Waxes Paraffin Glycerides	Protectors Coatings	They help to avoid drying or dehydration of the edible film providing flexibility.
***Additives***			
Plasticizers	Glycerol Aloe Resins	Viscosity Resistance Flexibility	They decrease the intermolecular force and the melting temperature in the mixture. They also modify the viscosity and the rheological properties.
Chaotropic agents	Urea	Destructuring agent	They increase the solubility of polymers in water.
Others	Polyphenols	Antioxidants Stabilizers Fungicides Herbicides Fertilizers	They work as stabilizers as well as protection for the products.

**Table 3:** Edible films containing different types of bioactive compounds and natural extracts.

Bioactive Compounds	Bio-Based Matrix	Additives	Functionality	Reference
Propolis extract	Cassava starch	Beeswax	Antimicrobial	[80]
Grape cane extract	Thermoplastic starch	Glycerol	Antifungal, antimicrobial	[81]
*Lactobacillus plantarum*, *Lactobacillus casei* subsp. *casei* and *Saccharomyces boulardii*	Gelatin and low methoxyl pectin	Glycerol	Probiotic	[82]
*Tricholoma terreum* extract	Chitosan	Glycerol and acetic acid	Antioxidant, antimicrobial	[83]
*Eriobotrya japonica* extract	Starch and banana peel flour	Glycerol	Antioxidant	[84]
Blackberry powder	Arrowroot starch	Glycerol	Antioxidant	[85]
Coconut water	Coconut protein precipitate	Glycerol	Antioxidant	[86]
Microencapsulated maltodextrin	Carboxymethyl cellulose	Glycerol and olive oil	Antioxidant	[87]
Prickly pear peel powder	Carboxymethyl cellulose	Glycerol	Antioxidant	[88]
Carrot β-carotenes	Cassava starch	Glycerol and sunflower oil	Antioxidant	[89]
Shrimp waste lipid extract	Gelatin	Glycerol	Antioxidant, anti-inflammatory	[90]
Lysozyme	Zein	Polyethylene glycol and ascorbic acid	Antioxidant, antimicrobial	[91]
Clove essential oil	Soy protein isolate and microfibrillated cellulose	Glycerol	Antioxidant, antimicrobial	[66]
Extracted spent coffee ground	Cassava starch	Polyvinyl alcohol and citric acid	Antioxidant, antimicrobial	[92]
Cinnamon oil	Soybean polysaccharide	Glycerol	Antioxidant, antimicrobial	[60]
Levofloxacin	Bacterial cellulose and pectin	−	Antimicrobial	[93]
Castor oil	Alginate	Glycerol	Antimicrobial	[67]
Nisaplin	Hydroxypropyl methylcellulose and nanofibrillated cellulose	Glycerol	Antimicrobial	[94]
Lysozyme nanofibers	Pullulan	Glycerol	Antimicrobial	[95]
Carvacrol	Halloysite nanotubes	Polypropylene	Antimicrobial	[96]
Bitter vetch protein	Mesoporous silica nanoparticles	Glycerol	Antimicrobial	[97]
Poly [2-(acryloyloxy) ethyltrimethylammonium chloride]	Chitosan	−	Antimicrobial	[98]
Clove, cumin, caraway, marjoram, cinnamon, and coriander essential oils	Alginate and montmorillonite	Glycerol and tween 80	Antimicrobial	[99]
Oregano essential oil	Citrus peel pectin	Glycerol	Antimicrobial	[100]
Clove, fennel, cypress, lavender, thyme, herb-of-the-cross, pine and rosemary essential oils	Chitosan and gelatin	Glycerol	Antimicrobial	[101]
Cinnamaldehyde, linalool, isoeugenol and citral	Ethylene-vinyl alcohol copolymer	−	Antifungal	[102]
Cinnamaldehyde and graphite	Chitosan	−	Antifungal	[103]

**Table 4:** Coatings with microorganism against pathogens applied in food models.

Biopolymeric Matrix	Additives	Food Type	Microorganisms	Inhibited Pathogens	Reference
Alginate	Glycerol	Ham slices	*Lactobacillus plantarum* and *Lactobacillus pentosus*	*Brochothrix thermosphacta*, *Pseudomonas* spp., Enterobacteriaceae, yeasts/molds and *Listeria monocytogenes*	[114]
Hydroxypropylmethyl cellulose, sodium caseinate, pea protein and corn starch	Glycerol	Grapes	*Candida sake*	*Botrytis cinerea*	[115]
Locust bean gum	−	Mandarins	*Wickerhamomyces anomalus, Metschnikowia pulcherrima* and *Aureobasidium pullulans*	*Penicillium digitatum* and *Penicillium italicum*	[116]
Alginate and locust bean gum	Glycerol	Oranges	*Wickerhamomyces anomalus*	*Penicillium digitatum*	[117]
Agar	Glycerol and green tea extract	Hake fillets	*Lactobacillus paracasei* and *Bifidobacterium lactis*	*Shewanella putrefaciens* and *Photobacterium phosphoreum*	[118]
Alginate and corn starch	Glycerol	Coated biscuits	*Lactobacillus plantarum*	*Salmonella, Escherichia coli* and *Streptococcus thermophilus*	[119]
Starch and alginate	Glycerol	Cold-smoked salmon covered	*Carnobacterium maltaromaticum*	*Listeria monocytogenes*	[120]
Alginate	Glycerol, palmitic acid and β-cyclodextrin	Strawberries	*Cryptococcus laurentii*	Mold	[121]

**Table 5:** Edible coatings-probiotics applied in food models.

Biopolymeric Matrix	Additives	Probiotic Microorganisms	Food Product	Survival Time	Reference
Maltodextrin, alginate and carboxymethyl cellulose	Glycerol	*Asparagus racemosus*	Chevon sausages	21 days	[128]
Hydroxypropylmethyl cellulose, sodium caseinate, pea protein and corn	Glycerol	*Candida sake*	Grapes	14 days	[129]
Methylcellulose	Sorbitol and citric acid	*Lactobacillus plantarum*	Apples	90 days	[130]
Alginate and whey protein	Glycerol	*Lactobacillus rhamnosus*	Bread	7 days	[131]
Carboxymethyl cellulose and alginate	−	Brewer yeast	Grapes	13 days	[132]
Gelatin and glucose	Sorbitol and cysteine	*Lactobacillus acidophilus* and *Bifidobacterium bifidum*	Hake fish	15 days	[133]
Corn starch	−	*Lactobacillus acidophilus*	Bread	24 h	[72]

**Table 6:** Edible films and coatings applied in food models.

Food Application	Biopolymeric Matrix	Additives	Coating Technique	Positive Results	Reference
***Fruits***					
Figs	Chitosan	Acetic acid, canola oil, cinnamon essential oil and Rosselle extract	Spread	Antioxidant capacity was preserved, color change was delayed and *Alternaria alternata* growth was inhibited	[138]
Bell pepper	Chitosan	Acetic acid, canola oil, glycerol and chitosan/α-pinene nanoparticles	Spread	Flavonoids and antioxidant capacity were not modified and *Alternaria alternata* growth got slow.	[139]
Papaya	Papaya puree and alginate	Glycerol and citric acid	Dipped	Shelf-life was extended	[59]
	Carrageenan	Glycerol and citric acid	Dipped	Ripening was delayed and shelf-life was extended	[140]
Blueberries	Alginate, chitosan, apple fiber and orange fiber	Glycerol, inulin and oligofructose	Dipped	Sensory quality was improved, and shelf-life was extended	[141]
	Chitosan, calcium caseinate, alginate and semperfresh^TM^	Glycerol and tween 20	Dipped	Ripening was delayed and flavor, texture and visual appearance were maintained	[142]
Strawberries	Chitosan	Acetic acid, canola oil, cinnamon essential oil and Roselle extract	Wrapped	Antioxidant capacity was increased, and shelf-life was extended	[143]
	Chitosan and beeswax	Glycerol and tween 80	Dipped	Quality was preserved and shelf-life was extended	[144]
	Chitosan and carotene-proteins	Glycerol and polyvinyl alcohol	Dipped	Microbial and fungal growth were controlled, and antioxidant activity was maintained	[145]
	Chitosan and chitosan nanoparticles	Glycerol, acetic acid and propolis extract	Dipped	Total phenols, flavonoids and antioxidant capacity were increased, ripening process was not modified, and sensory characteristics were not modified	[146]
	Fish gelatin and citrus pectin	Glycerol and hydroxytyrosol-3,4-dihydroxyphenylglycol	Dipped	Mold growth was delayed, and shelf-life was extended	[147]
	Cassava starch	Propolis extract	Dipped	Vitamin C content was promoted	[148]
Fresh-cut jackfruit bulbs	Xanthan, alginate and gellan gum	Glycerol and 1-methylcyclopropene	Dipped	Microbial growth was inhibited, and shelf-life was extended	[149]
Fresh-cut kiwifruit	Cactus pear mucilage	Glycerol and tween 20	Dipped	Visual quality and flavor were improved, and shelf-life was extended	[150]
Fresh-cut apples	Whey protein	Glycerol, citric acid and montmorillonite clay	Wrapped	Shelf-life was extended	[151]
	Carboxymethyl cellulose	Glycerol, calcium and acid ascorbic	Dipped	Vitamin C and antioxidant capacity were maintained	[152]
	Chitosan	−	Dipped	Quality was enhanced	[153]
	Alginate, gellan gum, pectin and apple fiber	Glycerol, ascorbic acid and inulin	Dipped	Quality was enhanced and shelf-life was extended	[154]
	Chocolate and milk butter	Polyglycerol polyricinoleate and ascorbic acid	Dipped	Anti-aging effect was produced	[155]
	Olive oil and sunflower oil	Lecithin and ascorbic acid	Spread	Anti-aging effect was produced	[155]
	Whey protein, soy protein, alginate and carrageenan	Glycerol	Dipped	Physical changes were controlled, and shelf-life was extended	[156]
	Cassava starch and carnauba wax	Glycerol and stearic acid	Wrapped	Physicochemical properties were improved	[157]
	Soybean gum, jojoba and Arabic gum	Glycerol and paraffin oil	Wrapped	Quality was maintained	[158]
Red grapes	Gelatin, corn starch and waxy maize starch	Glycerol and sorbitol	Dipped	Quality was enhanced and shelf-life was extended	[159]
Fresh-cut pineapple	Alginate	Glycerol, sunflower oil, lemongrass essential oil, calcium chloride, ascorbic acid, and citric acid	Dipped	Quality was preserved and shelf-life was extended	[160]
Fresh-cut mangoes	Alginate	Glycerol, sunflower oil, calcium chloride, ascorbic acid, and citric acid	Dipped	Browning agent was delayed, and shelf-life was extended	[161]
Fresh-cut watermelon	Alginate, pectin and calcium lactate	Glycerol	Dipped	Texture was preserved and shelf-life was extended	[162]
***Vegetables, Plants and Cereals***					
Saffron	Maltodextrin and nanocellulose	−	Spread	Physicochemical properties were improved	[163]
Potatoes	Locust bean gum	Glycerol	Dipped	Physical changes, microbial growth and to nutritional quality were controlled	[164]
Taro corms	Chitosan and starch	Glycerol	Dipped	Quality was enhanced, microbial growth was inhibited, and shelf-life was extended	[165]
Tomatoes	Citrus peel pectin	Glycerol and oregano oil	Spread	Antifungal effect was generated, and phenol content and antioxidant activity were increased	[166]
	Carnauba wax	Mineral oil	Spread	Antioxidant activity was increased	[167]
	Chitosan and zeolite	Tween 80 and acid lactic	Dipped	Ripening was delayed	[168]
	Soy protein, carboxymethyl cellulose and oleic acid	Glycerol, ascorbic acid and sodium benzoate	Dipped	Physical characteristics were enhanced, and shelf-life was extended	[169]
Cherry tomatoes	Hydroxypropyl methylcellulose and beeswax	Glycerol, tween 80 and oleic acid	Dipped	Growth fungal was reduced and physical appearance was maintained	[170]
	Hydroxypropyl methylcellulose and beeswax	Glycerol and oleic acid	Dipped	Growth of *Botrytis cinerea* was reduced and physical appearance was improved	[171]
Shiitake mushrooms	Alginate	Silver nitrate, sodium borohydride and polyvinylpyrrolidone	Dipped	Shelf-life was extended	[172]
Broccoli	Methylcellulose, polycaprolactone and alginate	Glycerol, tween 80, organic acids mixture, rosemary extract, Asian spice essential oil and Italian spice	Dipped	Growth of *Escherichia coli*, *Salmonella typhimurium* and *Listeria monocytogenes* was controlled	[173]
Spinach	Agar, κ-carrageenan, and konjac	Glycerol	Wrapped	Freshness was maintained and shelf-life was extended	[174]
White asparagus	Sodium carboxymethyl-cellulose, whey protein isolate and pullulan	Sucrose fatty acid ester, polyethylene glycol, sorbitol and stearic acid	Dipped	Weight loss was reduced, and quality was preserved	[175]
***Animal and Dairy Products***					
Sausages	Maltodextrin, alginate and carboxymethyl cellulose	Glycerol and Terminalia arjuna	Wrapped	Shelf-life was extended	[176]
	Gelatin and carrageenan	Glycerol, lard and beeswax	Dipped	Weight loss was reduced	[177]
Chicken meat	Mango peel powder, ciclodextrin and gelatin	Glycerol and polyvinyl alcohol	Wrapped	Shelf-life was extended	[178]
	Gum Arabic	Sorbitol, polyvinyl alcohol, and *Zanthoxylum rhetsa* extract	Wrapped	Bioactive compounds were increased, and shelf-life was extended	[179]
	Linear low-density polyethylene	Cinnamon essential oil and silver-copper	Wrapped	Antimicrobial capacity was increased, and shelf-life was extended	[180]
Butter	Low-density polyethylene	Yerba mate and carotenoid extracts	Wrapped	Antimicrobial and antioxidant capacities were increased, and shelf-life was extended	[181]
Ham slices	Cassava starch, chitosan and gallic acid	Glycerol	Wrapped	Shelf-life was extended	[182]
Fresh chicken breast	κ-Carrageenan and chitosan	Glycerol and oriental mustard extract	Dipped	*Campylobacter jejuni* was reduced and shelf-life was extended	[183]
Chicken nuggets	Alginate	Calcium chloride	Dipped	Microwave heating was improved	[184]
Bream fish	Alginate	Glycerol, vitamin C and tea polyphenols	Dipped	Bacterial growth was inhibited, and sensory values was enhanced	[185]
Cheese	Galactomannan and chitosan	Glycerol, sorbitol and oil corn	Spread	Shelf-life was extended	[186]
Poached turkey	Alginate, pectin, κ-carrageenan, starch, and xanthan gum	Nisin, novagard CB1, guardian NR100, sodium lactate, sodium diacetate and potassium sorbate	Dipped	Growth of *Listeria monocytogenes* was inhibited	[187]
***Bakery***					
Bread	Pectin, alginate and whey protein	Glycerol and tween 20	Sprayed	Moisture was decreased	[188]
	Starch	Glycerol and ε-poly-L-lysine	Wrapped	Shelf-life was extended	[189]
